# Supplementary material for: Mechanisms governing the pioneering and redistribution capabilities of the non-classical pioneer PU.1
Source: Nat Commun. 2020 Jan 21;11:402. doi: 10.1038/s41467-019-13960-2 (PMC6972792; doi:10.1038/s41467-019-13960-2)
Supplement: Supplementary file 7 — Source data [file 41467_2019_13960_MOESM7_ESM.zip › Source_Data/FigureS2/FigureS2B_MotifScanOutput/KmeansCluster6/homerResults/motif34.similar.html]

motif34

## Information for motif34

C
G
T
A
C
T
A
G
C
T
A
G
A
G
T
C
G
T
A
C
A
C
G
T
A
G
T
C
C
T
G
A
C
T
A
G
A
C
T
G
G
T
C
A
  
Reverse Opposite:  

A
C
G
T
A
G
T
C
G
A
T
C
A
G
C
T
C
T
A
G
C
G
T
A
A
C
T
G
C
T
A
G
A
G
T
C
A
G
T
C
C
G
A
T
  

|  |  |
| --- | --- |
| p-value: | 1e-31 |
| log p-value: | -7.258e+01 |
| Information Content per bp: | 1.925 |
| Number of Target Sequences with motif | 100.0 |
| Percentage of Target Sequences with motif | 3.04% |
| Number of Background Sequences with motif | 268.8 |
| Percentage of Background Sequences with motif | 0.62% |
| Average Position of motif in Targets | 80.8 +/- 76.3bp |
| Average Position of motif in Background | 106.1 +/- 75.9bp |
| Strand Bias (log2 ratio + to - strand density) | 0.5 |
| Multiplicity (# of sites on avg that occur together) | 1.00 |
| Motif File: | file (matrix) reverse opposite |

### Similar de novo motifs found

|  |  |  |  |  |  |  |  |
| --- | --- | --- | --- | --- | --- | --- | --- |
| Rank | Match Score | Redundant Motif | P-value | log P-value | % of Targets | % of Background | Motif file |
| 1 | 0.793 | C T A G C T A G A T C G C G T A T C A G C T A G A G T C G T A C A C G T A G T C C T G A C A T G | 1e-27 | -64.272247 | 2.46% | 0.46% | motif file (matrix) |
| 2 | 0.770 | G T A C A G C T T C A G C G T A A C T G T C A G A G T C G A T C C G A T A G T C A G T C G T A C G A T C | 1e-27 | -63.863725 | 2.82% | 0.62% | motif file (matrix) |
